# Nuclear Translocation of Crk Adaptor Proteins by the Influenza A Virus NS1 Protein

**DOI:** 10.3390/v8040101

**Published:** 2016-04-15

**Authors:** Leena Ylösmäki, Riku Fagerlund, Inka Kuisma, Ilkka Julkunen, Kalle Saksela

**Affiliations:** 1 Department of Virology, University of Helsinki and Helsinki University Hospital, 00014 Helsinki, Finland; leena.ylosmaki@helsinki.fi (L.Y.); riku.fagerlund@helsinki.fi (R.F.); inka.kuisma@helsinki.fi (I.K.); 2 Department of Virology, University of Turku, 20520 Turku, Finland and Virology Unit, Department of Infectious Disease Surveillance and Control, National Institute for Health and Welfare (THL), 00300 Helsinki, Finland; ilkka.julkunen@utu.fi

**Keywords:** NS1, influenza A virus, SH3 domain, Crk, virus-host interaction

## Abstract

The non-structural protein-1 (NS1) of many influenza A strains, especially those of avian origin, contains an SH3 ligand motif, which binds tightly to the cellular adaptor proteins Crk (Chicken tumor virus number 10 (CT10) regulator of kinase) and Crk-like adapter protein (CrkL). This interaction has been shown to potentiate NS1-induced activation of the phosphatidylinositol 3-kinase (PI3K), but additional effects on the host cell physiology may exist. Here we show that NS1 can induce an efficient translocation of Crk proteins from the cytoplasm into the nucleus, which results in an altered pattern of nuclear protein tyrosine phosphorylation. This was not observed using NS1 proteins deficient in SH3 binding or engineered to be exclusively cytoplasmic, indicating a physical role for NS1 as a carrier in the nuclear translocation of Crk. These data further emphasize the role of Crk proteins as host cell interaction partners of NS1, and highlight the potential for host cell manipulation gained by a viral protein simply via acquiring a short SH3 binding motif.

## 1. Introduction

Influenza A virus (IAV) belongs to the *Orthomyxoviridae* family of enveloped viruses. It has a segmented genome consisting of eight single stranded negative-sense RNA strands. The non-structural protein 1 (NS1) of IAV is an important virulence factor, and a remarkably multifunctional protein that acts in several different ways to facilitate IAV replication (for reviews, see [1,2]).

The dynamic localization of NS1 in the nucleus as well as in the cytoplasm of IAV-infected cells is mediated by two nuclear localization signals (NLS) and by one nuclear export signal (NES) [3,4,5]. Soon after IAV infection, newly synthesized NS1 accumulates in the nucleus, but at late time points of infection it is transported into the cytoplasm. The conserved NLS1 of NS1 protein involves the amino acids R35, R37, R38, and K41 [3,6], while NLS2 is virus strain-specific, and it is located in the C-terminus of the protein [3,6,7]. The NES is located between the amino acids 138–147, leucine residues 144 and 146 being critical for its function [8,9].

The NS1 protein has several reported functions both in the nucleus and in the cytoplasm. In the nucleus, NS1 can inhibit cellular mRNA maturation and export by interacting with cleavage and polyadenylation specificity factor (CPSF), poly(A)-binding protein II (PABPII), mRNA splicing machinery, and nuclear export factors [10,11,12]. In the cytoplasm, NS1 prevents the activation of interferon-inducing proteins by inhibiting RNA helicase retinoic acid inducible gene-I (RIG-I) through a direct interaction [13,14], and by preventing RIG-I ubiquitination via interacting with ubiquitin E3 ligases TRIM-25 and Riplet, [15,16]. NS1 also inhibits the activity of protein kinase R (PKR) [17], and 2′-5′-oligoadenylate synthetase (OAS) [18], two important interferon-induced antiviral proteins.

In addition, NS1 can activate the host cell phosphatidylinositol 3-kinase (PI3K) cascade, a signaling pathway intimately involved in viral replication and innate immunity, by interacting directly with p85β, a regulatory subunit of the PI3K complex [19,20]. PI3K activation is further enhanced by NS1 proteins that contain an SH3 binding motif, which mediates a strong and selective binding to the cellular adaptor proteins Crk (Chicken tumor virus number 10 (CT10) regulator of kinase) and Crk-like adaptor protein (CrkL) [21]. This NS1 SH3 binding motif is commonly found in avian IAVs, but only in some human IAV strains, including the 1918 pandemic Spanish flu virus. This potentiation of PI3K activation involves reorganization of the cellular p85β-Crk protein complex. While SH3 binding-incompetent NS1 proteins simply bind to p85β in this complex, PI3K-superactivating NS1 proteins hijack the SH3 domain of Crk, thereby breaking the pre-existing p85β-Crk complex and assembling an alternative trimeric complex where NS1 is a bridging factor between p85β and Crk [22].

Crk proteins consist of a family of three members: CrkI, CrkII, and CrkL. CrkII and CrkL both contain one SH2 and two SH3 domains, while CrkI is a truncated form of CrkII that due to an alternative mRNA splicing possess only the SH2 and the N-terminal SH3 domain [23,24]. Although Crk proteins lack any enzymatic activity, they play a crucial role in cell biology by serving as essential adaptor proteins linking together different signaling molecules, such as tyrosine kinases and small G proteins through their SH2 and SH3 domains. They coordinate numerous biological processes, ranging from cell proliferation, cell adhesion and migration, phagocytic and endocytic pathways, apoptosis, and regulation of gene expression (for reviews, see [25,26]). The SH2 and SH3 domains of Crk proteins are highly homologous and display similar binding preferences and they have several overlapping roles, for example, in maintaining the cell structure and motility in mouse embryonic fibroblast (MEF) cells [27]. Use of knockout mice has revealed also some non-overlapping roles for these proteins in embryonic development. Knockout of CrkI/II or CrkL individually leads to different developmental defects in mice and they die perinatally [28,29]. Most of the cellular functions described for Crk proteins involve coordination of cytoplasmic signaling processes. However, Crk proteins have also been reported to enter the nucleus to regulate additional signaling pathways involved in malignant transformation and programmed cell death. The nuclear partners for Crk proteins are not well known, but prominently include the tyrosine kinase c-Abl, whose nuclear functions are important in cellular responses to DNA damage, cell cycle progression, and apoptosis [30]. Moreover, nuclear translocation of CrkII and its interaction with the nuclear tyrosine kinase Wee1 has been reported to be proapoptotic [31,32]. It has also been reported that the binding of CrkL to phosphorylated form of signal transducer and activator of transcription (STAT5) leads to translocation of the complex into the nucleus where it binds to the promoter region of *c-Abl* or *Bcr-Abl* genes in chronic myeloid leukemia (CML) cells [33,34]. Regulation of the nuclear entry of Crk proteins is not well understood. CrkII and CrkL have a nuclear export signal located in theC-terminal SH3-domain [35], but all Crk proteins lack a canonical nuclear localization signal, and apparently they can enter the nucleus only through interaction with other proteins that contain a functional NLS [36].

Since both NS1 and Crk have distinct nuclear and cytoplasmic functions, and since the effects on cellular physiology described for nuclear Crk proteins appear to depend on interaction partners that are actively transported into the nucleus, we examined how NS1 might influence the intracellular distribution of Crk proteins. Here we report that infection of cells with IAV encoding NS1 proteins that are competent for Crk binding, in contrast to viruses encoding NS1 lacking the SH3 ligand motif, cause a robust translocation of Crk proteins from the cytoplasm into the nucleus, which is associated with a noticeable change in tyrosine phosphorylation pattern of proteins in the nuclear fraction.

## 2. Materials and Methods

### 2.1. Cell Culture

The human lung epithelial (A549) and the human hepatocellular carcinoma (Huh-7) cell lines were maintained in Dulbecco′s Modified Eagle Medium (DMEM) (Sigma Aldrich, St. Louis, MO, USA) supplemented with 4500 mg/L of glucose, 10% fetal bovine serum (FBS) (Gibco, Carlsbad, CA, USA), 0.05 mg/mL penicillin, 0.05 mg/mL streptomycin (Sigma Aldrich), and 1 mM L-glutamine (Sigma Aldrich) at 37 °C in 5% CO_2_.

### 2.2. Recombinant Influenza A Viruses

The recombinant influenza A viruses were generated by using a plasmid-based reverse genetics as previously described [37]. A/WSN/1933 IAV was used as the background virus. The NS segment originated from either A/WSN/1933/H1N1 or A/Mallard/Netherlands/12/2000/H7N3 virus. The codon changes to NS1 sequence (A/WSN T215P; A/Mallard K217E) were introduced using overlapping polymerase chain reaction (PCR) mutagenesis. Influenza A/WSN/1933 recombinant viruses were propagated in 11-day-old embryonated chicken eggs at 34 °C for three days. The recombinant viruses used in this study are: A/WSN-NS1^Mallard(wt)^, A/WSN-NS1^Mallard(K217E)^, A/WSN-NS1^WSN(wt)^, and A/WSN-NS1^WSN(T215P)^.

### 2.3. DNA Transfections and Plasmids

A549 and Huh-7 cells were transfected by using a Lipofectamine 2000 reagent (Invitrogen, Waltham, Massachusetts, USA) according to manufacturer′s instructions. The vector for A/Mallard myc-NS1 wild-type (WT) has been described before [21]. To generate fluorescent fusion proteins, mCherry was fused to the N-terminus of A/Mallard NS1, and enhanced green fluorescent protein (eGFP) to the N-terminus of CrkL. To generate a cytoplasmic A/Mallard NS1 (Cyto), the NES from MAPKK1 (LQKKLEELEL) was inserted between the mCherry and NS1 coding sequences. In addition, the NLS1 of NS1 protein was mutated (R38A, R41A) by standard overlap PCR mutagenesis. All plasmid constructs were verified correct by DNA sequencing.

### 2.4. Antibodies

The following primary antibodies were used in this study: mouse monoclonal anti-CrkL (clone 5–6, Millipore, Billerica, MA, USA), mouse monoclonal anti-Crk (clone 22, BD Transduction Laboratories, San Jose, California, USA), rabbit monoclonal anti-phospho Akt (Ser473) (D9E, Cell Signaling Technology, Danver, MA, USA), mouse monoclonalanti-α-tubulin (DM1A, Sigma-Aldrich), rabbit polyclonal anti-Histone H3 (Cell Signaling Technology), monoclonal mouse anti-phosphotyrosine (PY20, Santa Cruz Biotechnology, Dallas, Texas, USA), and guinea-pig polyclonal anti-NS1 [3]. The secondary antibodies for Western blotting were: IRDye680CW goat anti-mouse IgG, IRDye680CW goat anti-rabbit IgG, and IRDye800CW goat anti-rabbit, and IRDye800CW rabbit anti-guinea pig were from LI-COR Biotechnology (Lincoln, NE, USA). Secondary antibodies for immunofluorescence staining were: AlexaFluor 488 goat anti-guinea pig IgG (Abcam, Cambridge, UK), and AlexaFluor 546 goat anti-mouse IgG (Molecular Probes, Eugene, OR, USA). Nuclei were stained with Hoechst.

### 2.5. Immunoprecipitation and Detection

For immunoprecipitation A549 cells were infected with recombinant IAVs for 24 h, and the cells were collected and lysed in 1% NP40 lysis buffer (150 mM NaCl; 50 mM Tris–HCl, pH 7.9; 1% NP40). Cell lysates were used for immunoprecipitation with an anti-CrkL antibody coupled to Dynabeads protein G magnetic beads (Invitrogen). To examine the phosphorylation status of Akt, Huh7 cells on 6-well plates were transfected with 4 μg of plasmid DNA. Transfected cells were serum-starved for 12 h, and 48 h after transfection the cells were lysed in 1% NP40 lysis buffer. Western blots were visualized with the Odyssey infrared imaging system (LI-COR Biosciences, Lincoln, NE, USA).

### 2.6. Cell Fractionation

A549 cells were seeded on 10 cm diameter well plates at 3 × 106 density. The next day, the cells were mock infected or infected with recombinant IAVs at a multiplicity of infections (MOI) 2 in the presence of 5 μg/mL of N-alpha-tosyl-L-phenylalanyl chloromethyl ketone (TPCK)-treated trypsin (Sigma Aldrich). 24 h after infection the cells were scraped into 500 μL of ice cold Buffer A (20 mM Tris, pH 7.5, 100 mM NaCl, 300 mM sucrose, 3 mM MgCl2) supplemented with 0.5% Triton X-100. The cells were incubated on ice for 10 min and after that the nuclei were pelleted at 800 g for 10 min. The cytoplasmic extract (C) was collected and centrifuged at 16,100 g for 15 min. To prepare the nuclear extract (N), the nuclear pellet was washed once with Buffer A + 0.5% Triton X-100 and twice with Buffer A. The nuclei were suspended in 70 μl of Buffer B (20 mM·Tris, pH 8.0, 500 mM·NaCl, 2 mM·EDTA, pH 8.0, 0.1% Igepal) and sonicated for 3 s. The nuclear proteins were collected after centrifugation at 16,100 g for 15 min.

### 2.7. Immunofluorescence Staining and Confocal Imaging

For immunofluorescence microscopy, A549 cells were grown on coverslips and infected at an MOI of 0.5 in the presence of TPCK-treated trypsin (5 μg/mL). At 20 h after infection, the cells were fixed with ice cold methanol for 10 min at −20 °C, permeabilized with 0.1% Triton X-100, and incubated with guinea-pig anti-NS1 antibody, followed by AlexaFluor 488 goat anti-guinea pig IgG. CrkL was stained with mouse anti-CrkL antibody, followed by AlexaFluor 546 goat anti-mouse IgG. The cells were then examined with Leica TCS SP8 confocal microscope. Channels were scanned sequentially. The mean intensities of the CrkL fluorescence signal in the nuclei were analyzed by using the open source software, FiJi distribution of ImageJ (Version 1.50b, NIH) [38].

## 3. Results

### 3.1. SH3 Binding-Competent NS1 Proteins Translocate Crk Proteins into the Nucleus

**Figure 1:**
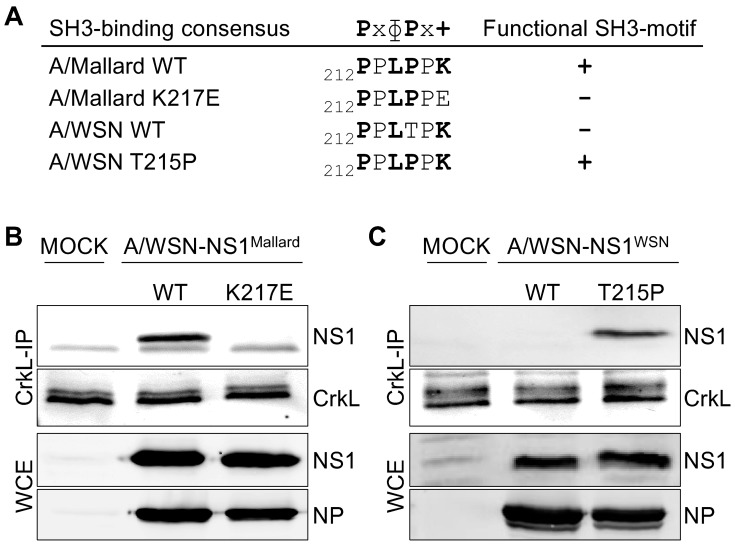
A functional SH3 binding motif in the non-structural protein-1 (NS1) is required for interaction with Crk-like adapter protein (CrkL) in influenza A virus (IAV)-infected cells. (**A**) The consensus sequence of class II SH3 binding motif, and its presence (**+**) or absence (**−**) in the C-terminal region (residues 212–217 shown) of NS1 proteins of the recombinant IAV strains used in this study. In SH3 binding consensus x indicates any residue, ɸ a hydrophopic residue, and + a positively charged amino acid, which for Crk-family SH3 domains is preferable a lysine residue; (**B,C**) Co-immunoprecipation of NS1 proteins with CrkL from lysates of A549 cells infected with recombinant A/WSN-based IAV strains expressing wild-type or mutant NS1 proteins derived from A/Mallard (**B**) or A/WSN (**C**) for 24 h at a multiplicity of infections (MOI) 2. Note that these NS1 proteins naturally differ in their SH3 binding capacity, and the mutations introduced in them thus have opposite effects. NS1 and nucleoprotein (**NP**) blots from whole cell extracts (**WCE**) before anti-CrkL immunoprecipitation are shown to control equal infection of the cells by the different viruses.

To study the Crk/NS1 interaction in an infectious setting, we generated a set of recombinant viruses using a typical human IAV A/WSN/1933/H1N1 (A/WSN) as a background strain. These recombinant viruses are isogenic with wild-type A/WSN virus, except for the segment 8 (NS segment), which encodes either the wild-type or a mutated NS1 from an avian IAV A/Mallard/Netherlands/12/2000/H7N3 (A/Mallard) or a mutant construct of NS1 of A/WSN. To generate an SH3 binding-incompetent mutant of the A/Mallard NS1, a K217E mutation was introduced into its NS1 sequence. Conversely, to engineer the naturally SH3 binding-incompetent A/WSN NS1 to become SH3 binding-competent, a T215P mutation was introduced into its NS1 sequence. Although not directly relevant for this study, it should be noted that the T215P mutation could also alter the phosphorylation pattern of NS1 as T215 has been reported as a functional phosphorylation site [39,40]. The mutations made in the NS1 sequence do not affect the NS2/NEP open reading frame (ORF). The sequences of the relevant SH3-binding regions in the NS1 proteins of these viruses are shown in Figure 1A.

To establish that the engineered mutations had the expected effects on the capacity of the corresponding A/Mallard and A/WSN NS1 proteins to interact with Crk proteins in IAV infected cells, we immunoprecipitated endogenous CrkL (Figure 1B,C) from mock infected or recombinant virus-infected A549 cells and examined NS1 co-precipitation by Western blotting. As seen in Figure 1B, while wild-type A/Mallard NS1 readily co-precipitated with CrkL, the NS1 mutant (K217E) did not associate with CrkL at detectable levels. Conversely, no association of wild-type A/WSN NS1 with CrkL could be detected, whereas efficient co-precipitation of the mutant NS1-T215P protein with a restored Crk SH3-binding motif was observed (Figure 1C).

**Figure 2:**
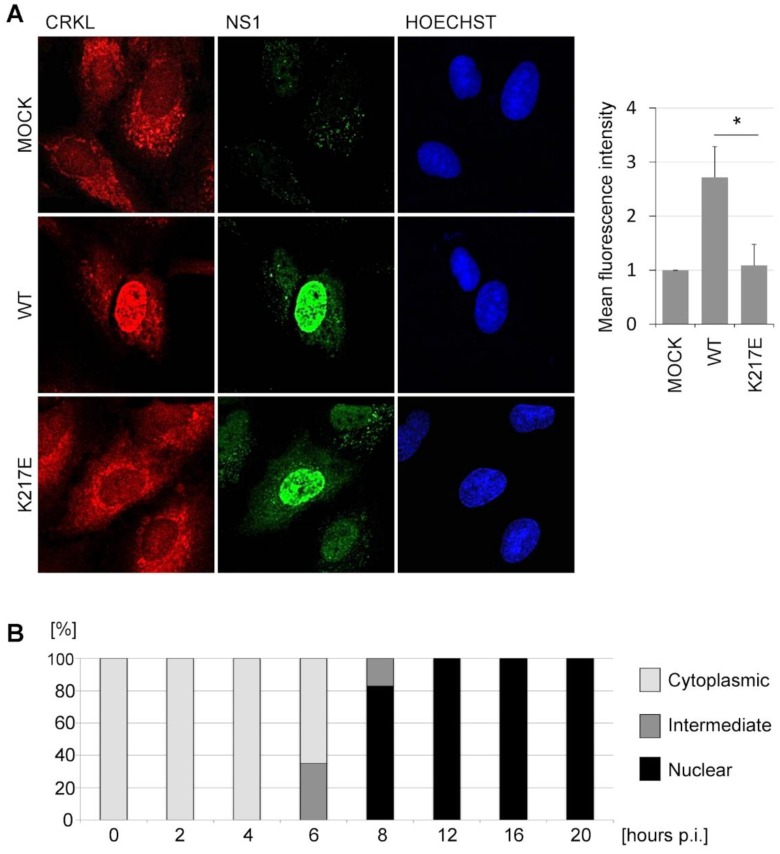
Infection of cells with IAV expressing SH3 binding-competent NS1 causes nuclear relocalization of CrkL. (**A**) Immunofluorescence staining of NS1 and CrkL in A549 cells that were mock-infected (upper panel) or infected with A/WSN-NS1^Mallard(wt)^ (middle panel) or A/WSN-NS1^Mallard(K217E)^ (bottom panel) for 20 h at a MOI 0.5. The nuclei were visualized by staining with Hoechst. The mean intensity of CrkL fluorescence in the nuclei was quantified from 50 cells infected with A/WSN-NS1^Mallard(wt)^ or A/WSN-NS1^Mallard(K217E)^ that also stained positive for positive for NS1, and was normalized to the mean fluorescence intensity of CrkL immunostaining of 50 mock-infected cells. The standard error is presented in the figure. The statistical significance of the differences was determined by Student′s t-test (* *p* < 0.001); (**B**) A549 cells were infected with A/WSN-NS1^Mallard(wt)^ for different time points at a MOI 0.5. The localization of CrkL was scored from 100 cells as a cytoplasmic, an intermediate, or a nuclear pattern.

Next, we analyzed the localization of NS1 and Crk proteins in the infected cells by immunofluorescence staining and confocal imaging. A549 cells were infected with an MOI of 0.5 with A/WSN-NS1^Mallard(wt)^ or A/WSN-NS1^Mallard(K217E)^ recombinant viruses and the cells were fixed 20 h later. CrkL was localized mainly in the cytoplasm in mock-infected cells, and only faint staining was observed in the nucleus (Figure 2A, top row). In the infected cells, both the WT and the K217E-mutant NS1 proteins were predominantly localized in the nucleus (Figure 2A, in green). Strikingly, in cells infected with A/WSN-NS1^Mallard(wt)^ CrkL was found to mainly co-localize with NS1 in the nucleus (Figure 2A, middle row), whereas in cells infected with A/WSN-NS1^Mallard(K217E)^ the distribution of CrkL was indistinguishable from its predominantly cytoplasmic localization pattern in mock-infected cells (Figure 2A, bottom row). Very similar differential distribution was also observed for Crk when examined by immunostaining with an antibody that detects both CrkI and CrkII (data not shown). To more formally establish this effect, the mean intensity of CrkL-fluorescence signal in NS1-positive nuclei was quantified from 50 individual cells infected with A/WSN-NS1^Mallard(wt)^, A/WSN-NS1^Mallard(K217E)^, or mock-infected cells (Figure 2A, right panel). When the mean intensities of CrkL immunostaining in these nuclei were normalized to the value of the mock-infected cells, a robust and highly significant nuclear translocation of CrkL by SH3 binding-competent but not by SH3 binding-incompetent NS1 could be demonstrated.

When the kinetics of nuclear translocation of the Crk proteins was examined in more detail, we could observe first signs of nuclear accumulation of the Crk and CrkL at 6 h post-infection (p.i.) coinciding with the nuclei becoming clearly positive for NS1 staining (Figure 2B). At 8 h p.i. nuclear accumulation of Crk proteins was already prominent, and at 12 h p.i. Crk/CrL localization seemed already complete showing a pattern that looked identical to the 20 h p.i. time point shown in Figure 2A.

**Figure 3:**
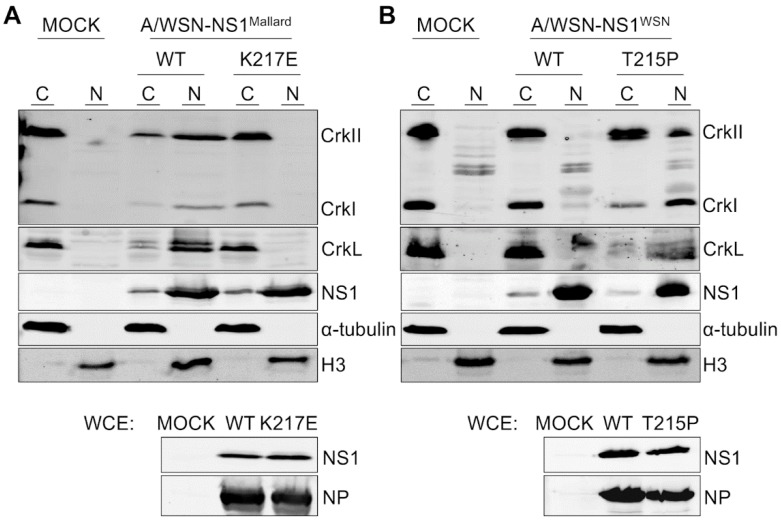
Nuclear translocation of CrkI, CrkII, and CrkL by SH3 binding-competent NS1 proteins demonstrated by subcellular fractionation. (**A**) Western blot analysis of cytoplasmic (**C**) and nuclear extracts (**N**) prepared from A549 cells that were mock-infected (**MOCK**) or infected for 24 h with recombinant A/WSN containing either the wild-type (**WT**) or the K217E mutant NS1 from A/Mallard virus at an MOI 2. In addition to antibodies against the Crk-family proteins and NS1, the blotted A549 fractions were also probed with antibodies against Histone H3 and α-tubulin to confirm successful separation of nuclear and cytoplasmic fractions. In addition, unfractionated whole cell extracts (**WCE**) of the infected cells were Western blotted with anti-NS1 and anti-NP antibodies to confirm uniform infection of cells by the different viruses; (**B**) Same as (**A**) except that the cells were infected with recombinant A/WSN virus carrying wild-type (**WT**) or the T215P mutant NS1 from A/WSN.

To extend and support these imaging studies by using a biochemical approach, we prepared cytoplasmic and nuclear fractions of cells infected with recombinant viruses and compared the presence of Crk proteins and wild-type or mutant NS1 proteins in these fractions (Figure 3A,B). The quality and purity of the nuclear and cytoplasmic protein fractions obtained from these cells were established by Western blotting of these preparations using antibodies against anti-α-tubulin (a cytoplasmic marker) and anti-histone H3 (a nuclear marker).

As expected, NS1 protein was seen mainly in the nuclear fractions regardless of the recombinant virus that was used to infect these cells. The nuclear fractions of mock-infected cells did not contain detectable amounts of CrkI, CrkII, or CrkL proteins (Figure 3A,B), whereas strong signals of expected size for these proteins were observed in the cytoplasmic fractions. Nuclear *vs.* cytoplasmic fractionation of Crk proteins derived from cells infected with viruses expressing an SH3 binding-incompetent version of NS1 (A/WSN-NS1^WSN(wt)^ and A/WSN-NS1^Mallard(K217E)^) was identical with that observed with mock-infected cells (Figure 3A,B). In sharp contrast, in cells infected with viruses having an SH3 binding-competent NS1 (A/WSN-NS1^WSN(T215P)^ and A/WSN-NS1^Mallard(wt)^) all Crk proteins could be abundantly detected in the nuclear fractions, especially CrkL becoming predominantly nuclear (Figure 3A,B).

### 3.2 NS1-Induced PI3K-Activation does not Depend on Crk Relocalization into the Nucleus

Our previous studies have shown that simultaneous recruitment of Crk proteins by NS1 substantially potentiates NS1-induced activation of PI3-kinase pathway [21,22]. While these signaling interactions would be expected to take place in the cytoplasm, it is nevertheless possible that subsequent nuclear transit of the bulk of cellular Crk proteins by NS1 could somehow contribute to the observed PI3K superactivation.

To address this possibility we generated a mutant NS1 protein that remains predominantly in the cytoplasm (NS1-Cyto). This was achieved by mutating the N-terminal NLS1 of A/Mallard NS1 (this strain does not contain NLS2) at the critical basic residues (R38A,R41A) combined with the addition of a strong heterologous nuclear export signal (NES) from mitogen-activated protein kinase kinase-1 (MAPKK1) [41].

**Figure 4:**
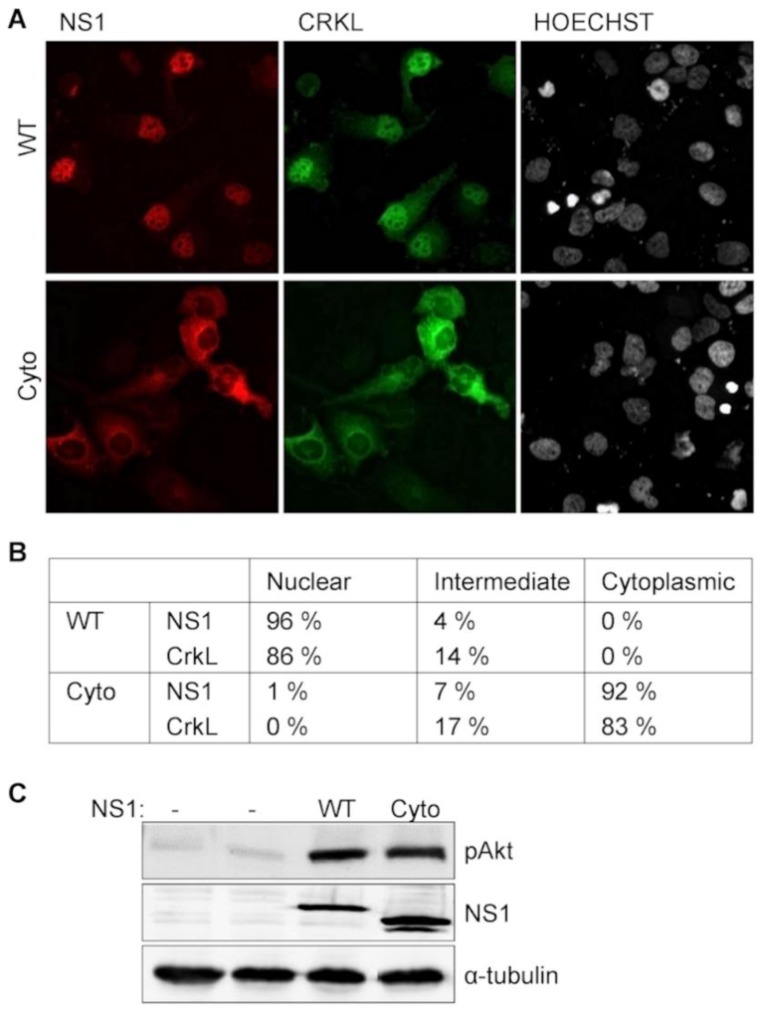
NS1-mediated Crk relocalization is independent of NS1-activated PI3K-signaling. (**A**) Fluoresence microscopy imaging of Huh7 cells co-transfected with eGFP-fused CrkL (green fluorescense) together with mCherry-fusion protein (red fluorescence) of wild-type A/Mallard NS1 (WT) or its dominantly cytoplasmic mutant NS1-Cyto; (**B**) Localization of NS1 and CrkL was examined in 100 cells from (**A**) and the observed fluorescence patterns were scored as nuclear, intermediate, or cytoplasmic; (**C**) PI3K-activation by wild-type and mutant version of NS1 revealed by Akt phosphorylation. Huh7 cells were transiently transfected with a vector expressing the indicated NS1 variants, and 48 h later examined by Western blotting with antibodies against phospho-Akt (pAkt), NS1, and α-tubulin.

The localization of NS1-Cyto was compared with wild-type A/Mallard NS1 by transient transfection of red fluorescent fusion protein (mCherry) derivatives of these NS1 proteins. Similar to the NS1 immunostaining in IAV-infected cells, in virtually all productively NS1-transfected cells (96%; of 100 cells counted) the red fluorescence of wild-type NS1 showed a distinctly nuclear localization pattern as illustrated in Figure 4A (upper panel). By contrast, only 1% of cells transfected with NS1-Cyto showed such a nuclear fluorescence, and in almost all (92%) of these cells the nuclei were devoid of NS1 signal and appeared as dark areas inside cytoplasmic red fluorescence (Figure 4A, lower panel), thus establishing the success of our double mutation approach to generate an NS1 mutant restricted to a cytoplasmic localization. Co-transfection of a vector expressing CrkL tagged with eGFP recapitulated our results obtained by infection with recombinant IAV variants, showing a prominently nuclear green fluorescence that faithfully co-localized with wild-type NS1. Conversely, in NS1-Cyto-transfected cells also CrkL fluorescence was found predominantly in the cytoplasm (see Figure 4B for statistics of the observed NS1 and CrkL localization patterns). When eGFP-CrkL was transfected alone (data not shown), a localization pattern similar to that observed for endogenous CrkL by immunostaining (Figure 2A).

When the capacity of NS1-Cyto to trigger PI3K-activation in transfected cells was compared with that of wild-type A/Mallard NS1, a similar increase in the phosphorylation of Akt, a downstream effector of the PI3K cascade was observed (Figure 4C). Thus, while recruitment of Crk proteins by NS1 is required for the enhancement PI3K-activation [21,22], NS1-mediated nuclear translocation of Crk is not. Likewise, it can be concluded that while the cytoplasmic interaction between NS1 and Crk is sufficient to potentiate PI3K activation, nuclear targeting of Crk cannot be triggered by a contact with NS1 in the cytoplasm, but indeed physically depends on the nuclear entry of Crk-NS1-complex driven by the NLS of NS1.

### 3.3. A Change in Nuclear Protein Tyrosine Phosphorylation after NS1-Mediated Nuclear Re-Localization of Crk

**Figure 5:**
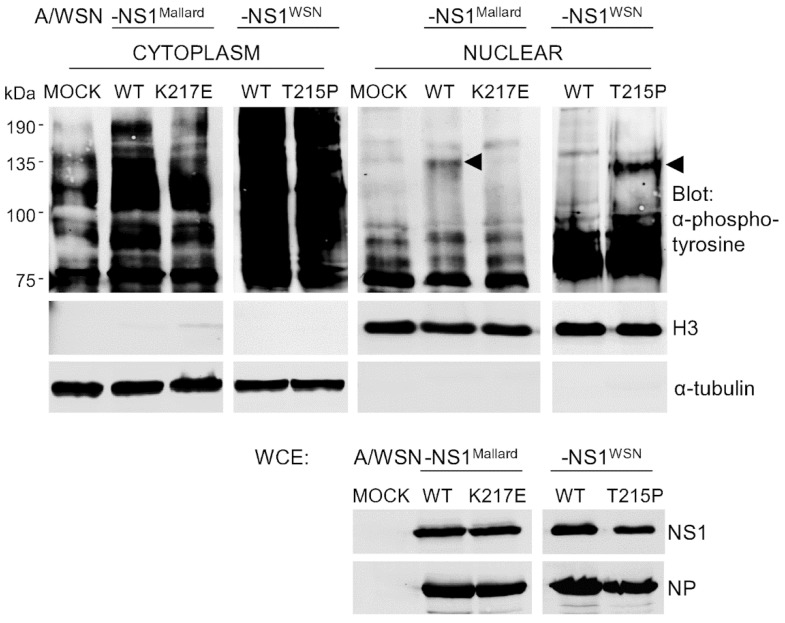
Nuclear targeting of Crk by NS1 causes a change in the nuclear protein tyrosine phosphorylation pattern of IAV-infected cells. A549 cells were infected with recombinant viruses as indicated for 24 h at a MOI 2, and treated with pervanadate for 10 min before cytoplasmic (**C**) and nuclear (**N**) extracts were prepared. The extracts were probed with an anti-phosphotyrosine antibody. As in Figure 3, shown are also H3 and α-tubulin blots to verify the quality of the subcellular fractionation, as well as blotting of whole cell extracts (WCE) with antibodies for NS1 and NP to verify equal NS1 expression of NS1 and uniform infection of cells with the different viruses.

Crk proteins interact with many tyrosine phosphorylated proteins as well as tyrosine kinases [25], and upon the original discovery of the viral Crk oncogene (v-Crk) an increase in cellular protein tyrosine phosphorylation was described as a hallmark of Crk-mediated malignant transformation [42]. To study whether NS1-mediated nuclear translocation of Crk proteins in IAV-infected cells would lead to any functional consequences, we compared the patterns of protein tyrosine phosphorylation in nuclear extracts of A549 cells that were mock-infected or infected for 24 h with IAV expressing NS1 proteins either capable (A/WSN-NS1^Mallard(wt)^ and A/WSN-NS1^WSN(T215P)^) or not capable (A/WSN-NS1^WSN(wt)^ and A/WSN-NS1^Mallard(K217E)^) for binding and nuclear targeting of Crk. To enhance the accumulation of phosphotyrosine-modified proteins, the cells were treated for 10 min with the phosphotyrosine phosphatase-inhibitor pervanadate before they were fractionated into nuclear and cytoplasmic extracts that were subjected to Western blotting with an anti-phosphotyrosine (anti-pTyr) antibody (Figure 5). Successful subcellular fractionation was confirmed by probing with antibodies against prototypic nuclear and cytoplasmic proteins, and uniform infection of the cells was demonstrated by probing unfractionated lysates of these cells with antibodies against IAV NS1 and NP. While the nuclear extracts of cells infected with viruses expressing NS1 proteins lacking Crk binding activity did not differ from mock-infected cells in their patterns of tyrosine phosphorylated proteins, a prominent new phosphotyrosine-containing protein with a MW of about 135 kDa appeared in the nuclear extracts of cells infected with A/WSN-NS1^Mallard(wt)^ or A/WSN-NS1^WSN(T215P)^ (Figure 5, pointed with arrows). Thus, we conclude that Crk proteins translocated into the nucleus upon IAV infection via their binding to NS1 can reprogram cellular signaling pathways in the nucleus as evidenced by altered nuclear protein tyrosine phosphorylation.

## 4. Discussion

Acquiring a target motif for an SH3 domain-mediated interaction provides a convenient strategy for viruses to hijack key signaling pathways that regulate the behavior of their host cells. The high-affinity Crk SH3 binding site in the carboxyterminus of the IAV NS1 protein is an interesting example of the ease of such virus-host interaction evolution. As highlighted by the recombinant IAV strain A/WSN-NS1^WSN(T215P)^ used in this study, a single nucleotide change in the segment 8 of the viral genome to change an ACT codon into CCT is sufficient to give rise to an NS1 protein with a capacity for fundamentally altering host cell physiology by taking the control of Crk-dependent signaling pathways. The degree of this control can be quite remarkable as evidenced by the dramatic relocalization of cellular Crk proteins to the nucleus described in this study.

Despite our present findings as well as other effects on the host cell previously assigned to the Crk SH3 interaction motif of NS1 [21,22,43,44], the overall role of SH3 binding capacity of NS1 in supporting IAV replication and pathogenesis remains unclear. Should this property alone provide a clear-cut replicative or immune evasion advantage, it would quickly become fixed in IAV evolution also in humans, which has not happened. Mutations analogous to our A/WSN-NS1^WSN(T215P)^ mutant of the human IAV strains A/Udorn/72 and the 2009 Swine Flu pandemic virus (A/California/04/09) to introduce an SH3 binding site in NS1 did not result in enhanced viral replication [40,45]. On the other hand, another human IAV strain, A/PR8/8/34, was shown to benefit from the introduction of an SH3 binding motif to its NS1 and was more pathogenic in mice [46]. It is tantalizing to note that the 1918 pandemic Spanish flu virus A/Brevig Mission/1/18/H1N1 is one of the known human IAV strains that naturally contains this sequence motif, suggesting a positive contribution to viral fitness in this context [21]. It is likely that the utility of hijacking Crk signaling depends on a complex combination of other functional variables of IAV that are not only determined by the sequence variation in the multifunctional NS1 protein itself, but also encoded by other segments of its genome.

In addition to enhancing PI3K activation [21] via re-organization of the PI3K-Crk-complex [22], a functional Crk SH3 binding site of NS1 has previously been linked to suppression of c-Jun N-terminal kinase-activating transcriptional factor 2 (JNK-ATF2) pathway [43] and to an inhibition of the tyrosine kinase c-Abl [44]. Our current data suggest that this list may have to be extended to include many more of the diverse cellular functions of the Crk protein family. It should mentioned, however, that the interferon-antagonizing effects of NS1 do not fall into this category, and have been shown to be independent of Crk SH3 binding [21].

Nuclear relocalization of the bulk of cellular Crk proteins can be expected to affect several cytoplasmic Crk functions. However, phosphorylation and protein interactions are probably more relevant in regulation of these functions than the total cytoplasmic Crk concentration. This would explain why we did not observe the PI3K-NS1-Crk complex-dependent enhancement of PI3K activity in cells expressing an NS1 mutant that was forced to remain cytoplasmic and thus unable to move Crk into the nucleus. Perhaps more important than the reduction in the amount of cytoplasmic Crk may indeed be the triggering of new signaling events in the nucleus induced by NS1-mediated nuclear transportation of Crk.

Previous studies on cancer biology have described triggering of major signaling events and outcomes caused by nuclear transport of Crk proteins. CrkII has been reported to participate in apoptosis by activating caspases and binding to the nuclear cell cycle regulator, Wee1 through CrkII SH2 domain [31,32]. On the other hand, CrkL has been reported to bind via its SH2 domain to tyrosine phosphorylated Stat5 [34,47]. The complex can translocate into the nucleus to bind Stat5-responsive elements followed by regulation of gene expression [48,49]. It will be interesting to see how closely NS1-mediated nuclear relocalization of Crk recapitulates these events, and to what extent the complex with NS1 redirects Crk to alternative nuclear protein complexes and functions.

As a demonstration that NS1-mediated nuclear transport of Crk proteins can indeed reprogram nuclear signal transduction pathways, we showed the appearance of a novel nuclear tyrosine phosphorylated protein with an estimated molecular weight (MW) of 135 kDa (pp. 135). However, despite the established role of Crk proteins in regulating cellular pTyr protein levels, it should be noted that we cannot exclude the possibility that some SH3-dependent function of NS1 other than the observed robust nuclear transport of Crk could account for the associated changes in nuclear protein tyrosine phosphorylation.

The 135 kDa size of the novel pTyr-decorated nuclear protein matches with the major cytoplasmic tyrosine-phosphorylated protein partner of Crk proteins, the p130Cas [50,51]. However, while this remains a possible scenario, so far we have not been able to prove that cytoplasmic p130Cas is transported into the nucleus as a part of an NS1-Crk-p130Cas complex. Another candidate for pp. 135 that we have considered, but likewise not been able to prove is c-Abl, a partially nuclear [52] tyrosine kinase that can be activated by Crk [53] and undergo autophosphorylation [54]. However, since Crk uses its N-terminal SH3 domain for binding to c-Abl [55], this interaction could take place only after dissociation of the NS1-Crk complex following its nuclear entry. At any case, further studies on the identity of pp135 as well as comprehensive analyses on the changes in the nuclear phosphoproteome induced by NS1-mediated nuclear transport of Crk proteins in IAV-infected cells clearly warrants further experimental attention in order to better characterize the functional significance of this novel function of NS1.

IAV is an unusual RNA virus in the sense that it replicates in the nucleus of the host cell. Thus, it is easy to understand why manipulation of the nuclear environment would be relevant for promoting the IAV life cycle. Ludwig and colleagues have reported that activation of the apoptotic effector caspase-3 at late stages of the IAV replication cycle is required for efficient nuclear exit of viral RNP complexes [56]. Given the previous reports on the capacity of nuclear Crk protein to promote apoptosis [31,32], a role of NS1-mediated nuclear transport of Crk in facilitating vRNP release from the nucleus poses one potentially interesting possibility. Since lamins are important caspase substrates and key components of the nuclear lamina, we have initiated studies on lamin cleavage and integrity of the nuclear lamina during IAV infection. Our preliminary data suggest that viruses expressing Crk binding-competent NS1 proteins could indeed induce lamin A/C cleavage and induce more extensive changes in the nuclear morphology than do viruses with NS1 proteins that lack the SH3 binding motif [57].

The present results further emphasize the role of Crk proteins as host cell interaction partners of IAV, although much work remains to be done to characterize the detailed nuclear functions of Crk protein relocalization to the nucleus by NS1, and the significance of this reprogrammed signaling for IAV replication and pathogenesis. Nevertheless, the remarkable potential of SH3 binding-competent NS1 proteins to robustly relocate a key family of host cell signaling factors from the cytoplasm to the nucleus attests to the extensive consequences that adopting a short protein interaction motif by a viral protein can have, and suggests that the role of the NS1-Crk interaction in cell biology of IAV may be broader than we have so far appreciated.
